# Experiences of pregnant Venezuelan migrants/refugees in Brazil, Ecuador and Peru: a qualitative analysis

**DOI:** 10.1186/s12884-024-06334-0

**Published:** 2024-02-23

**Authors:** Michele Zaman, Victoria McCann, Sofia Friesen, Monica Noriega, Maria Marisol, Susan A. Bartels, Eva Purkey

**Affiliations:** 1 School of Medicine, Queen’s University, Kingston, ON Canada; 2 Department of Public Health Sciences, Queen’s University, Kingston, ON Canada; 3 International Organization for Migration, Panama City, Panama; 4 International Organization for Migration, Pacaraima, Brazil; 5 Department of Emergency Medicine, Queen’s University, Kingston, ON Canada; 6 Department of Family Medicine, Queen’s University, Kingston, ON Canada

**Keywords:** Migration, Pregnancy, Global health, South America

## Abstract

**Background:**

It is estimated that since 2014, approximately 7.3 million Venezuelan migrants/refugees have left the country. Although both male and female migrants/refugees are vulnerable, female migrants/refugees are more likely to face discrimination, emotional, physical, and sexual violence. Currently there is a lack of literature that explores the experiences of pregnant Venezuelan migrants/refugees. Our aim is to better understand the experience of this vulnerable population to inform programming.

**Methods:**

In the parent study, Spryng.io’s sensemaking tool was used to gain insight into the gendered migration experiences of Venezuelan women/girls. A total of 9339 micronarratives were collected from 9116 unique participants in Peru, Ecuador and Brazil from January to April 2022. For the purpose of this analysis, two independent reviewers screened 817 micronarratives which were identified by the participant as being about someone who was pregnant, ultimately including 231 as part of the thematic analysis. This was an exploratory study and an open thematic analysis of the narratives was performed.

**Results:**

The mean age and standard deviation of our population was 25.77 ± 6.73. The majority of women in the sample already had at least 1 child (62%), were married at the time of migration (53%) and identified as low socio-economic status (59%). The qualitative analysis revealed the following main themes among pregnant Venezuelan migrants/refugees: xenophobia in the forms of racial slurs and hostile treatment from health-care workers while accessing pregnancy care; sexual, physical, and verbal violence experienced during migration; lack of shelter, resources and financial support; and travelling with the hopes of a better future.

**Conclusion:**

Pregnant Venezuelan migrants/refugees are a vulnerable population that encounter complex gender-based and societal issues that are rarely sufficiently reported. The findings of this study can inform governments, non-governmental organizations, and international organizations to improve support systems for pregnant migrants/refugees. Based on the results of our study we recommend addressing xenophobia in health-care centres and the lack of shelter and food in host countries at various levels, creating support spaces for pregnant women who experience trauma or violence, and connecting women with reliable employment opportunities and maternal healthcare.

**Supplementary Information:**

The online version contains supplementary material available at 10.1186/s12884-024-06334-0.

## Background

### Venezuelan migration context

 In the last five decades, the number of international migrants/refugees has been steadily increasing globally [1]. Venezuela, in particular, has been experiencing an intense and rapid outflow of migrants/refugees which is unprecedented in Latin America. The International Organization for Migration (IOM) states that this is the largest external displacement crisis in Latin American history [2]. As of June 2023, it is estimated that there are 2.48 million Venezuelan migrants/refugees in Colombia, 449, 678 in Brazil, 502 214 in Ecuador, 1.50 million in Peru, and 444 423 in Chile [3]. These neighbouring countries have faced significant challenges, socially and politically, in responding to the massive influx of migrants/refugees [4]. Peru, Ecuador, Colombia, and Brazil have rolled out important regularization strategies; however, there are still enormous challenges to regularization and socioeconomic integration in most countries in Latin America and the Caribbean [5, 6].

The Venezuela migration crisis is complicated and multifaceted. The mass migration patterns can be directly and indirectly linked with the country’s current economic, political, public health and human rights crisis [4]. The R4V Coordination Platform for Refugees and Migrants from Venezuela (R4V Platform) estimates that since 2014, approximately 7.3 million Venezuelans have left the country [3]. Once a rich country, Venezuela is now stricken with poverty and hunger. For instance, between 2013 and 2021 Venezuela lost more than 75% of its Gross Domestic Product (GDP) [6, 7], the highest GDP loss for any country not at war in the last 50 years [7, 8]. The COVID-19 pandemic exacerbated the country's economic crisis, and in 2020 more than 95% of Venezuelans were living below the poverty line [7, 8]. Venezuela has had the highest hyperinflation rate in the world [9]. It is estimated that consumer prices had increased 13,379 percent during 2018, and in 2019 the hyper-inflation rate was approximately 10 million percent, creating widespread poverty [9, 10]. Additionally, healthcare and public health infrastructure has deteriorated, resulting in higher infant mortality, maternal mortality, infectious diseases transmission, and vaccine-preventable disease rates [4]. Between 2000 and 2015, Venezuela saw a 359% increase in malaria cases, dengue incidence increased more than 4 times from 1990 to 2016, and there is active transmission of Chagas disease occurring within communities [11]. Human trafficking and violence have also increased in recent years [4].

### Pregnancy & migration

Although both male and female migrants/refugees are vulnerable, female migrants/refugees are more likely to face discrimination, emotional, physical, and sexual violence [12]. Furthermore, health status can also magnify a migrant or refugee’s vulnerability [13]. This is particularly true during pregnancy. A woman's health is sensitive to change during pregnancy and previous studies have found that pregnant migrants/refugees are more vulnerable in that they are likely to have poorer pregnancy outcomes, and are at a greater risk for maternal and neonatal morbidity [12, 14–16]. They are also more likely to have less social support, to experience violence, and are more dependent on the host country's societal characteristics such as legal system and health institutions compared to their pregnant native counterparts [17].

A systematic review that analyzed epidemiological studies comparing pregnancy outcomes in native and immigrant women in European countries from 1966 to 2004 concluded that immigrant women had poorer pregnancy outcomes [18]. The data showed that immigrant births were: 43% higher risk of low birth weight, 24% of preterm delivery, 50% of perinatal mortality, and 61% of congenital malformations [18]. Poor pregnancy outcomes were significantly reduced in countries with strong integration policies for migrants [18]. Another systematic review that explored maternal health care in immigrant populations also concluded that immigrant groups had higher incidence of stillbirths and neonatal death, increased risk for maternal death and depression, reduced access to healthcare facilities and lack of communication with healthcare providers [19]. A focus group in Switzerland interviewed Portuguese and Turkish migrant communities and found that stress related to living conditions, heavy workload, insufficient communication with healthcare providers, and feelings of racism and discrimination from society negatively affected their pregnancy experience [20]. Pregnant migrant women have a different health profile than their pregnant native counterparts [21]. The intersection between pregnancy and migration amplifies the challenges associated with a woman's health, introducing a dynamic interplay that demands a nuanced examination [22].

The Venezuelan migration crisis continues at an unprecedented magnitude, resulting in a public health and humanitarian crisis, yet little is known about the experiences and needs of vulnerable migrant/refugee populations, including those who are pregnant. There is a paucity of literature that examines the experiences of Latin American pregnant women, especially while these women are en route in their migration journey. As a result, in the present study we propose to answer the following research question: what are the experiences of pregnant Venezuelan women during migration? Our aim is to identify the challenges (including safety, accessible health care, and financial hardship, among others) faced by pregnant migrants/refugees in order to inform programming, services, and policies that will address their unique needs.

## Methods

The current work was a secondary analysis of data derived from a larger mixed-methods, cross-sectional study implemented in Ecuador, Peru, and Brazil in January to April 2022 by the International Organization for Migration (IOM) and Queen’s University in Canada. The parent study’s purpose was to examine gendered threats for migrating Venezuelan women/girls, including various forms of GBV, in order to strengthen protection interventions while also improving support programs to better meet refugees’/migrants’ needs. Theresearch team consists of a gender expert (MN), a Venezuelan migrant (MM), two medical students (MZ and VM), a public health PhD student (SF), and two physician/public health researchers (EP and SAB). Full details of the study implementation have been previously published and will be summarized here [23].

### Sensemaking approach

Sensemaking is a mixed-methods, narrative capture approach that facilitates the collection and interpretation of brief narratives on a topic of interest (in this case, the migration experiences of Venezuelan women/girls) [24–26]. We refer to the resultant brief narratives as ‘micronarratives’ since they are typically shorter than transcripts from in-depth qualitative interviews. Uniquely, sensemaking empowers research participants to interpret the experiences shared in their micronarratives by responding to a series of analytical questions [22], which reduces interpretation bias and allows for more nuanced mixed-methods data. The primary distinguishing feature of a sensemaking approach is that the research participant shares a brief narrative in response to a broad, open-ended prompting question and then interprets the experience themselves.

In the present study, we used Spryng.io’s sensemaking platform to gain insight into the gendered migration experiences of Venezuelan women/girls. Participants were asked to share a migration experience using one of three micronarrative prompts (Supplementary 1). Participants audio-recorded their migration experience and then interpreted the experience by responding to a series of pre-programmed questions about the events shared. Multiple-choice questions at the end of the survey collected further contextualizing data (e.g., who was the woman/girl in the shared experience, was the experience positive/neutral/negative, was the shared experience about a pregnancy) as well as socio-demographic information. Using a sensemaking approach, Spryng.io links the quantitative survey results to the qualitative micronarratives, creating a more comprehensive understanding of participants' experiences.

### Sensemaking survey

The sensemaking survey was developed in collaboration with partners from the IOM and from community-based organizations providing services to Venezuelan migrants/refugees (Supplementary 1). It was created in English, translated to Spanish, and then independently back-translated to confirm accuracy. The survey was pilot-tested with 25 Venezuelan women prior to data collection. Results from the pilot were used to improve question relevance and language clarity.

### Participants and data collection

Female and male self-identified Venezuelan migrants/refugees, aged 14 and older, were eligible to participate. Although the micronarrative prompts asked about the migration experiences of Venezuelan women/girls, males were also included (although not in this particular analysis), and they typically shared the migration experiences of their pregnant wives, partners, sisters, and daughters. The inclusion of men was particularly relevant for the parent study, which examined gendered migration experiences, including gender-based violence (GBV) because it is essential to engage with men on this issue. Furthermore, including men offers unique insights into cultural dynamics and potential intervention points and provides a more diverse range of perspectives to enrich our understanding of migration experiences. For the present analysis we used a gender-specific lens and only included micronarratives from women to gain a nuanced understanding of the unique challenges, coping mechanisms, and vulnerabilities of pregnant women during the migration process. This allowed for a more comprehensive exploration of this specific group's journey and experiences. Between January and April 2022, data were collected in three locations in each of Brazil (Pacaraima, Boa Vista, and Manaus), Peru (Tumbes, Lima, and Tacna), and Ecuador (Tulcàn, Manta, and Huaquillas). Potential participants were approached and invited to participate in the study in public spaces such as border crossings, markets, points of aid distribution, transportation depots, and shelters, creating a convenience sample. While IOM’s points of contact with refugees/migrants were included, the recruitment was much broader than this in order to collect more diverse experiences and perspectives (i.e., the sample extended well beyond those who were accessing support from IOM). All surveys were facilitated by a team of 19 Spanish-speaking research assistants (twelve females and six males), many of whom were Venezuelan. Co-author MM was a member of the interview team in Brazil. Before beginning data collection, all research assistants completed a three-day training which included topics such as sensemaking methodology, research ethics, prevention of sexual exploitation and abuse, and psychological first aid. The information letter and informed consent highlighted for potential participants that the research was about the migration experiences of Venezuelan women/girls with the goal of mitigating risks and improving services and programs to better support them. All data were collected in Spanish using handheld tablets out of earshot of others. The audio files ranged in length from less than a minute to more than 10 min. Surveys were completed privately unless the participant wished to have someone with them. Micronarratives were transcribed and translated from Spanish to English using the Sonix.ai software. We did not collect data about individuals who chose not to participate and surveys were delivered at a single point in time.

### Micronarrative selection

**Fig. 1:**
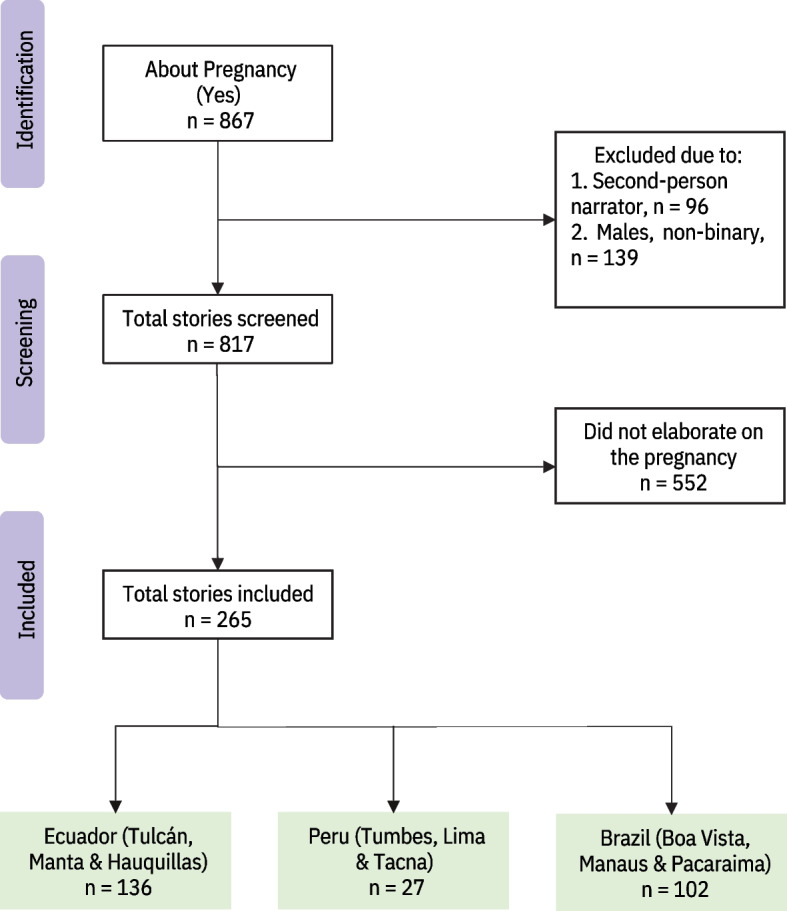
Micronarrative selection process flow diagram

In total, 9339 micronarratives were collected from 9116 Venezuelan migrants/refugees. A multiple-choice question in the survey asking whether the micronarrative was about pregnancy was used to identify the subsample for this analysis (*n* = 867). Additional micronarratives were identified through manual screening and a key word search that included pregnant, pregnancy, miscarriage, miscarry, abortion, and birth, adding another 185 micronarratives to the dataset. This particular analysis focused exclusively on first-person experiences among pregnant women and so third-person micronarratives (*n* = 96) and micronarratives shared by men or non-binary individuals (*n* = 139) were removed. Two trained reviewers (MZ and VM) independently screened the 817 micronarratives excluding those that did not elaborate on the pregnancy. Figure 1 is a flow diagram that outlines our micronarrative selection process.

### Analysis

Ultimately, 265 included micronarratives were reviewed independently by MZ and VM. Micronarratives were coded by both MZ and VM line-by-line using NVivo software. Since this was an exploratory study, an open thematic analysis was performed [27]. Every line of each narrative was grouped into categorical nodes and then nodes were grouped together to form themes. Examples of nodes were: humiliation, physical violence, pregnancy loss, hunger, dangerous trails, and shelter. Additionally, in our qualitative analysis, we reached a point of saturation, where no new themes or insights emerged from the coding, indicating a comprehensive understanding of the migration experiences among pregnant Venezuelan women. Discrepancies were resolved through discussion between the two coders and an arbitrator (EP) was involved if consensus was unable to be reached.

### Validation through community engagement

Using a community-centered approach, we shared preliminary results with community members and service providers during a series of workshops and focus group discussions in July 2022 (7 workshops with 120 service providers in total plus 14 focus group discussions with a total of 116 Venezuelan refugees and migrants). These discussions played a vital role in verifying facts and confirming that our findings were interpreted correctly and presented in a culturally appropriate manner. The methodology followed the COREQ checklist for reporting on qualitative studies which can be found in our supplementary information.

### Ethics

Informed consent was obtained from each participant and data were confidential since no identifying information was collected. No compensation was offered for participating since the survey was brief (12—15 min). Through IOM and partners, referral mechanisms were in place for any participant who required medical services including post-sexual assault care, psychosocial support, or immediate aid such as food or shelter. Ethics approval was obtained by Queen’s University Health Sciences and Affiliated Teaching Hospitals Research Ethics Board (#6,029,400).

## Results

### Demographics

A total of 265 micronarratives were included in our analysis and Table 1 presents the demographic characteristics of participants. The mean age and SD of our population was 25.77 ± 6.73. A majority of participating pregnant migrants/refugees already had at least 1 child (64%), and while most were married (52%), there were also many single parents (single/never married = 42% and divorced = 4%). Most participants noted that their socio-economic status was poor (57%) or average (27%). The 265 participants were from different parts of Venezuela. The most common locations were: Bolívar (17%), Anzoátegui (12%), Aragua (6%), La Guaira (3%), and Caracas D.F. (10%).

**Table 1:** Demographic information

	**Boavista**	**Huaquilla**	**Lima**	**Manuas**	**Manta**	**Tumbes**	**Tulcán**	**Tacna**	**Pacaraima**	**Total**
**Sample Size (N)**	**48**	**58**	**6**	**18**	**44**	**9**	**34**	**12**	**36**	**265**
**Age (mean)**	26.1 ± 8.19	29.0 ± 5.38	28.3 ± 5.95	28.4 ± 8.89	25.1 ± 6.24	24.4 ± 7.93	44.8 ± 6.69	38.7 ± 6.22	34.1 ± 5.84	25.77 ± 6.73
**Number of children, n (%)**										
0	2 (4%)	4 (7%)	-	-	-	1 (11%)	2 (6%)	1 (8%)	9 (25%)	19 (5%)
1–2	24 (50%)	41(71%)	6 (100%)	12 (66%)	28 (64%)	7 (78%)	21 (62%)	9 (75%)	21 (58%)	169 (64%)
3 or more	22 (46%)	12 (21%)	-	6 (34%)	16 (36%)	1 (11%)	11 (32%)	2 (17%)	4 (11%)	74 (30%)
Prefer not to say	-	1 (1%)	-	-	-	-	-	-	2 (6%)	3 (1%)
**Marital Status, n (%)**										
Married	35 (74%)	26 (45%)	2 (33%)	14 (78%)	16 (36%)	5 (56%)	15 (44%)	8 (67%)	18 (50%)	139 (52%)
Divorced	3 (6%)	1 (2%)	-	2 (11%)	-	2 (22%)	1(3%)	1 (8%)	1 (3%)	11 (4%)
Single	10 (20%)	29 (51%)	3 (50%)	2 (11%)	27 (61%)	2 (22%)	18 (53%)	3 (25%)	17 (47%)	111 (42%)
Prefer not to say	-	2 (3%)	1 (17%)	-	1 (3%)	-	-	-	-	4 (2%)
**Relative Wealth, n (%)**										
Very Poor	13 (27%)	6 (10%)	2 (33%)	3 (17%)	4 (9%)	-	3 (9%)	-	6 (17%)	37 (14%)
Poor	25 (52%)	42 (72%)	2 (33%)	5 (3%)	25 (57%)	5 (56%)	26 (76%)	3 (25%)	17 (47%)	151 (57%)
Average	10 (21%)	9 (15%)	2 (33%)	10 (56%)	13 (30%)	4 (44%)	5 (15%)	9 (75%)	10 (28%)	72 (27%)
Wealthy	-	1 (1%)	-	-	1 (2%)	-	-	-	-	1 (0.3%)
Prefer not to say	-	-	-	-	1 (2%)	-	-	-	3 (8%)	4 (2%)
**Length of Displacement, n (%)**										
< 1 year	23 (48%)	13 (21%)	2 (33%)	3 (17%)	1	5 (56%)	18 (52%)	1 (8%)	26 (72%)	98 (37%)
1–3 years	16 (33%)	36 (65%)		12 (66%)	18 (41%)	3 (33%)	10 (30%)	3 (25%)	4 (11%)	102 (38%)
3–5 years	9 (19%)	9 (15%)	4 (64%)	3 (17%)	16 (36%)	1 (11%)	6 (18%)	8 (67%)	4 (11%)	60 (23%)
> 5 years	-	-	-	-	3 (7%)	-	-	-	-	3 (1%)
Prefer not to say	-	-	-	-	-	-	-	-	2 (6%)	2 (1%)
**Venezuela Home, n%**										
Bolívar	19 (40%)	2 (3%)	1 (16%)	4 (22%)	-	-	1 (3%)	-	18 (50%)	45 (17%)
Anzoátegui	12 (25%)	4 (7%)	-	1 (6%)	2 (5%)	-	4 (12%)	2 (16%)	8 (22%)	33 (12%)
Aragua	1 (2%)	7 (12%)	1 (16%)	1 (6%)	2 (5%)	1 (11%)	3 (8%)	1 (8%)	-	17 (6%)
La Guaira	-	2 (3%)	1 (16%)	-	2 (5%)	1 (11%)	1 (3%)	1 (8%)	-	8 (3%)
Caracas D.F	-	4 (7%)	-	-	14 (31%)	1 (11%)	4 (12%)	3 (26%)	1 (3%)	27 (10%)
Other	16 (33%)	39 (68%)	3 (50%)	12 (66%)	24 (54%)	6 (67%)	21 (62%)	5 (42%)	9 (25%)	135 (50%)
Health issues* (Yes%)	3 (6%)	2 (3%)	1 (17%)	2 (11%)	0	2 (22%)	3 (9%)	0	2 (6%)	15 (6%)
**Did the micronarrative mention contraception? (%Yes)**	2 (4%)	3 (5%)	0	1 (5%)	4 (9%)	0	0	1 (8%)	7 (19%)	18 (7%)

^*^ Health issues = disability, depression, alcohol use

Our qualitative analysis revealed 4 main themes: (1) xenophobia while trying to access pregnancy care, (2) violence experienced during migration, (3) lack of shelter, resources, and financial support, and (4) travelling with the hopes of a better future.

### Theme 1 – xenophobia when trying to access pregnancy related medical care

There were many instances of xenophobia and humiliation described in the micronarratives. In fact, many women experienced xenophobic behavior from healthcare workers such as racial slurs and had trouble accessing adequate pregnancy care upon arrival to their destination countries.

*“The doctor always treated me badly, I heard him call me ‘Veneca’ , I asked them to stop this but they didn’t listen to me either. They told me that I was only good at complaining and that all of us ‘Venecas’ came to have children here to have a visa…Many people may not be affected by those comments, but they made me feel very bad, I even felt guilty for being pregnant and that I did not deserve to be pregnant in Ecuador, it was very difficult.” [Huaquillas, Ecuador, 27-years-old, 16866 SID]*

Pregnant women were treated badly in healthcare settings because the staff and administration sometimes shared beliefs that all Venezuelan women were only in their country to give birth to their children and thereby claim citizenship. They are often referred to as “Veneca” which is a racial slur against Venezuelans. Participants also described experiences where healthcare staff treated them with hostility.

*“I had to go through a very difficult situation in a clinic… despite the fact that I went with the money to give birth, they did not want to attend to me, they told me that people like me only came to give birth here to obtain nationality illegally and steal. They looked at me with the utmost contempt as if I were garbage.” [Huaquillas, Ecuador, 35-years-old, 16866 SID]*

The xenophobia experienced from healthcare workers sometimes put women at risk of life-threatening situations, exacerbated their vulnerability, and affected the quality of health care received. Many narratives described medical negligence which risked harm to their child and themselves.

*“I had an earlymiscarriage…the trip I took caused me harm…After finally arriving here, I felt a lot of pain and I started bleeding. They called an ambulance and it took me to a maternity ward…When I got there, they took me to the Emergency unit, but the doctor and the nurse treated me very badly. They checked and had blood on their fingers and concluded that nothing was wrong with me…They asked me to stand up and did not help me at all. I was in too much pain. It was hard for me to stand, it was hard for me to walk, it was hard for me to do everything, and they didn’t help me at all… And they said nothing was wrong with me that I should leave. I continued bleeding, and they practically did not attend to me.” [Boavista, Brazil, 23-years-old, 21230 SID]*

A common theme of distressing encounters with healthcare professionals marked by derogatory comments and discriminatory attitudes emerged from the micronarratives. The stories also illustrate the harmful stereotypes present in host countries which can result in refusal of care and negligence. These experiences compromise the emotional well-being of women and also creates a significant barrier to women accessing the necessary maternal healthcare they need.

### Theme 2 – GBV experienced during migration

Pregnant women faced violence and cruelty during migration in their destination countries, including verbal abuse, sexual abuse, and physical assaults with some being extreme enough to cause miscarriages. These violent experiences often led to humiliation and shame. Fear is also common during migration and it was intensified for pregnant women because they worried about their child’s safety and well-being in addition to their own.

*“Arriving in Colombia I was eight months pregnant with my baby. I was with my nephew and son. As we were coming down from one of the buses, people started throwing stones at us and one of these stones fell on my belly and another fell on my son…Thank god my nephew was not hurt but one hit my son and hurt his little head. My belly was hit but thank God nothing happened to me but my son has a scar on his forehead. They started shouting that they want Venezuelans out of their country, that they don't want Venezuelans here” [Huaquillas, Ecuador, 26-years-old, 21293 SID]*

Women shared stories of sexual violence they experienced during their migratory journey from men who were in positions of power such as police officers and employers. Women often experienced barriers to accessing abortions for resulting unplanned pregnancies and subsequently, many faced mental health and physical challenges. Women also experienced extortion to perform sexual acts through withholding pay, causing fear and anxiety of being fired with no financial support. Intimate partner violence was common. Many women relied heavily on their partners for emotional and physical support. They described the fear for their unborn child during these altercations.

*“When I tried to cross the border to Ecuador, the Ecuadorian police officers were offering to let me cross the border if I had sex with them or I had to give them a certain amount of money that I didn’t have…When I arrived at Peru, the Peruvian police offered me the same. They grabbed me and took me to a room. They had a little carpet there and they wanted to sexually abuse me. I started screaming and myself and another person there were able to escape from them. We waited until late at night and were able to crossover to Peru. When we were going through the desert to Arica in Chile, we were received and mistreated by the army, we tried to escape at night and the same night the marines did the same things to us. They told us that the Peruvians or Chilean will not help us because we are ‘shitty’ migrants, that we are very useless, and we arrive at the countries to mine their economy, stability, and country in general. When I arrived in Chile I found out that I was pregnant… I was released from the job I got in Chile because I was pregnant. When I tried to cross again to get to Tacna, the police and Carabineros [Chilean law enforcement agents] started to mistreat me even when I told them that I was pregnant. They pushed me, they mistreated me, they insulted me with a lot of nasty names… I arrived at Tacna, the Peruvian police extorted me. I told them I was pregnant and nonetheless they took my bags and they said I had to give them all the money I have. They collected 120 dollars from me” [Tacna, Peru, 27 years-old, 16265 SID]*

This devastating experience recalled by a young Venezuelan woman illustrates how micronarratives revealed a disturbing prevalence of violence experienced by women throughout the migration journey. Physical violence, including sexual violence and verbal abuse jeopardizes the safety of women and further compounds the vulnerabilites of this population.

*“In 2021 I re-entered before giving birth, again with the hope that I would do well this year. When I arrived here, I had a hard time getting a job. I did not find anything, and the places that would offer me a job, my bosses blackmailed me so that I would have sex with them, or they would not pay me.” [Manta, Ecuador, 22-years-old, 15692 SID]*

These micronarratives provide an insight to the tapestry of challenges faced by women while migrating and highlight both the inherent structural issues in their migration journey but also the deep rooted GBV vulnerabilities. GBV was a recurring theme throughout the stories. Pregnant Venezuelan women are vulnerable to physical harm, emotional distress, and economic exploitation.

### Theme 3 – lack of shelter, resources and financial support

Many pregnant women described the difficulties getting to their destinations after weeks of travel only to find no shelter and/or food available in the host community. It was common for participants to sleep on the streets when they initially arrived, and they were often hungry with no food or money. Shelters were often either unavailable due to lack of infrastructure and social support systems or the existing shelters were full. Other times the shelters were unsafe for women due to the risk of sexual or physical violence occurring in them.

*“When I arrived from Venezuela, I was eight months pregnant. I came with my seven-year-old son and my husband. We went through many hardships, we were robbed, they took our money, we had nothing to eat…In the journey we experienced lots of rain, lots of insects. We had nowhere to stay.” [Manaus, Brazil, 33-years-old, 17282 SID]*

*“I arrived here walking while pregnant, it was a tough experience because we slept on the street. We went for days without eating.” [Tulcán, Ecuador, 33-years-old, 22679 SID]*

Sadly, sleeping on the streets is mentioned in numerous micronarratives. The lack of housing and poor living conditions forces women into precarious living situations further jeopardizing their safety.

Lack of shelter caused significant distress. Pregnant migrants/refugees not only had to worry about their own well-being but that of their children and unborn children as well. Without safe shelter, women were forced into unsafe environments, which created fear and anxiety. These emotions were sometimes heightened by rumours of children being stolen and women being kidnapped and mistreated on the streets.

*“I was 7 months pregnant and had to make the journey here to Pacaraima. I stayed on the street with my two children. Sleeping on the street, on a mat. From there we stayed in the road network and we got a tent to sleep in…we really didn't have anywhere to stay…we didn’t have money, we had nothing for ourselves. It was dangerous because suddenly people would arrive to set up tents at night. I was afraid because I heard stories about children being stolen. I slept there with one eye open. I came to PTRIG [the screening post], and thank God I was given shelter.” [Boavista, Brazil, 35-years-old, 22975 SID]*

Financial concerns were also commonly expressed, with many women finding themselves in a new country with nowhere to sleep and no money to take care of themselves or their children. They described the difficulties of obtaining and keeping a job while pregnant. A few women who were employed found that they were paid less and treated differently because of their pregnancy. This resulted in a lot of emotional distress and physical hardship. Women described sleeping on the streets and going hungry for an extended period of time because it was also difficult to find work in host countries. In many stories women also raised concerns about having no option other than to engage in sex work in order to support themselves and their families.

*“ I came to Lima with my 2 year old son and I was pregnant with Vere. I couldn’t get a job and we had to return to Ecuador. But we couldn't find work there either; what we did was beg and sleep on the street. They called my husband to come back to Lima for a job interview. We really went through a lot of hardships. We went hungry, slept in the street, were cold, we spent a whole day walking with the children.” [Lima, Peru, 22-years-old, 14780 SID]*

The lack of economic opportunities for migrants creates a significant financial constraint, often hindering women from accessing essential needs such as healthcare and nutrition.

*“My biggest fear was having to prostitute myself to be able to eat because I left with little money from there and unfortunately my fear came true because we ran into bad people, they wanted to charge me double because I was 5 months pregnant.” [Huaquillas, Ecuador, 27-years-old, 16354 SID]*

Micronarratives depict the pervasive fear of engaging in prostitution as a survival strategy against economic hardships.

*“I have been in Peru for one year and I got pregnant and did not retire from my job because I needed to earn money for rent, food and sending money to Venezuela. And at the place where I worked, they lowered my salary when they realized I was pregnant.”[Lima, Peru, 26-years-old, 16260 SID]*

Food shortages and scarcity are a struggle many migrants express, adding to the many challenges migrants already face as they have to secure adequate nutrition for themselves, their unborn child and most times their other children they are travelling with as well.

The stories told by Venezuelan women shed light on the emotional toll from the extreme hardships women have to endure as they migrate. These include homelessness, hunger, and economic discrimination. The compounding effects of the lack of financial support and infrastructure available to migrating pregnant women makes them exceptionally vulnerable.

### Theme 4 – travelling with hopes for a better future

Many pregnant participants described how the physical and emotional hardships of migration were worth it due to the possibility of a better future for their families. They discussed the complicated situation in Venezuela that they were leaving behind in hopes of providing their children with a better quality of life. Additionally, many women described their hopes of going to a new country where their children would be able to pursue opportunities such as education that were not possible in Venezuela.

*“We are a family of 4 people. We came here to Brazil because of the many difficulties in Venezuela. In my case, when I came to Brazil, I was seven months pregnant. That was one of my strongest reasons for coming to Brazil. In Venezuela there is no possibility of a pregnant woman to work or have things for her baby. We decided to come here for a better future for our children and for the baby on the way. It was not easy at all, but the effort was worth it.” [Manuas, Brazil, 35-years-old, 21516 SID]*

The pursuit of stability, access to employment, educational opportunities and the hopes of being able to provide for their families in a way they were unable to prior to migration emerge as common threads in our micronarratives, highlighting the transformative potential migration has for pregnant Venezuelan women. The above quote is representative of the sentiments of many other women. The micronarratives also underscore the resilience of this population and the effort that is undertaken to create a better life for their families and themselves.

## Discussion

Literature on the experiences of pregnant migrants/refugees in South America is scarce. Our team was privileged to learn about the experiences of pregnant Venezuelan migrants/refugees, providing insights into the types of challenges pregnant women struggle with while migrating.

In summary, we identified 4 key themes including (1) women facing xenophobia while accessing pregnancy related care; (2) experiencing violence such as physical, sexual, and verbal abuse during the migration process; (3) the hardships of migrating without secured shelter, resources or financial support; and (4) migrating with the hope of a better future for themselves and their children.

With respect to healthcare, our study highlights the discrimination women endured attempting to access care whereas existing literature focuses primarily on the lack of sexual and reproductive healthcare available and the lack of contraception. Pregnancy is a time of high need in terms of access to appropriate nutrition, preventative medical care, and medical assessment and intervention. In 2016 the World Health Organization (WHO) revised their recommendations from four to eight as the minimum number of antenatal visits each woman should have to ensure a healthy pregnancy [28]. In addition, 49 recommendations were made to support nutrition, maternal and fetal assessment, preventative measures, support for common symptoms of pregnancy, and health system interventions. The purpose of these recommendations is to decrease the rates of maternal and neonatal morbidity, and to ensure an effective transition from pregnancy to labor and childbirth, and eventually positive parenting experience [29]. At the time these recommendations were made, it was reported that only 64% of pregnant women attained the previously recommended four antenatal visits [29]. While this may make recommending eight seem aspirational, these are important recommendations and interventions to improve maternal and child health. The women in our study, who struggled to access food and housing, let alone antenatal care, are unlikely to come close to meeting these recommendations. A study by Makuch et al., in 2021 looked at the reproductive health of Venezuelan women at the north western border of Brazil through 12 focus group discussions with 111 Venezuelan migrants/refugees [30]. Themes identified from this study were i) access to long-term reversible contraceptive was difficult to attain and ii) traditional gender imbalances make it difficult for women to protect themselves against sexually transmitted disease and human immunodeficiency virus [30]. Another study conducted by Bahamondes et al., in 2020 explored maternal health amongst Venezuelan migrants/refugees at the Brazilian border. This study discussed the intrinsic vulnerability of women in crisis situations that is compounded by pregnancy leading to increased risk of negative sexual and reproductive health outcomes [31]. Through interviews with 405 migrants/refugees, they found that the most common issue was the lack of access to contraceptive methods [31]. Interviewees overall were satisfied with the attention received at the maternity hospital, however 24% of the study population did not receive any prenatal or postnatal care [31]. Previous studies have also documented the lack of sexual health and reproductive services available and the poor obstetrical outcomes for migrating Venezuelan women [32–35].

In addition to challenges previously identified, our data provides insights into xenophobia in healthcare settings. A publication by IOM states that the discrimination Venezuelan migrants/refugees women experience in Colombia, Ecuador and Peru is a multifactorial phenomenon [35]. They propose that there are 3 main groups that experience high levels of discrimination: 1) LGBTQ + , 2) women who identify as Afro-Venezuelan and mestizo, and 3) women with irregular migration status [36]. Our findings suggest that pregnancy is a factor which also makes Venezuelan women more susceptible to discrimination. This is consistent with the construct of demographic anxiety, or the fear that one group of generally “new” or “different” people will increase to the extent that they will threaten national identity or unity. In this case, the high numbers of migrating Venezuelans likely trigger this fear in host populations at baseline, and this idea would be further embodied by the pregnant migrant who, as illustrated in our micronarratives, may be perceived to be migrating during pregnancy explicitly to claim citizenship for their unborn child. Around the world where migration and evolving birthrates occur, this demographic anxiety often leads to discrimination and prejudice with real implications for the health, well being, and integration of migrants [37].

Furthermore, past literature on female Venezuelan migrants/refugees also found that violence was commonplace. Makuch et al.,concluded that for migrant/refugee Venezuelan women, psychological threats, physical aggression and xenophobia was part of their everyday [38]. Rates of violence inside shelters were high and women had a hard time coping with these incidents [38]. Another study by Blukaz et al., discussed that GBV is particularly common for migrating Venezuelan women [35]. GBV can be defined as “violence directed against a person because of that person’s gender, or violence that affects persons of a particular gender disproportionately” [36]. Women are vulnerable while crossing borders due to the power imbalances between themselves and the border security with transactional sex occurring commonly and often unspoken of [9, 35, 39]. The violence experienced specifically by pregnant Venezuelan women is not documented in the scientific literature, a gap which this study seeks in part to fill. Our findings suggest that pregnant Venezuelan women are also struggling with high rates of violence during the migration process. Pregnant women are in a vulnerable state both physically and emotionally, and our micronarratives illustrate the fear women feel for themselves and their unborn child.

Consistent with our findings, IOM published a report which stated that the challenges most migrants/refugees faced while travelling to their destinations included lack of financial resources, food security, and lack of place to sleep [40]. Another report by the Centre for Migration Studies wrote that the humanitarian aid raised for the Venezuelan migration crisis is less than 1/10th of funds available per person for the Syrian refugee crisis [41]. The R4V Platform estimates that an additional $775 million billion dollars in funding is needed for non-governmental, charity and local organizations to provide adequate food, shelter, and medicine to migrants/refugees in host countries [42].

Lastly, many women from our study shared stories of emigrating with the hopes of finding a better future for themselves and their children. Previous studies on the Venezuelan migration crisis have written about how the worsening socioeconomic conditions and the deteriorating public health infrastructure is a major factor pushing individuals to emigration [41]. Individuals in Venezuela struggle with poverty, hunger, lack of maternal care and opportunities. Migrants/refugees take the risks associated with leaving their home to find a better life [41]. There is minimal written in academic literature about women who are pregnant in particular struggling with the challenges of migrations.

## Limitations

Results must be interpreted within the context of the study’s limitations. First, we used a convenience sample, and while there was a large number of respondents, it is possible that key groups were missed. Although the results cannot be generalized, we believe that by engaging with refugees/migrants in public locations rather than relying solely on points of service delivery, the sample is more diverse and inclusive. Second, sensemaking micronarratives tend to have less detail and depth than traditional qualitative interviews and as a result important ideas and experiences may have been omitted. However, we attempted to mitigate this by engaging with Venezuelan community members through a series of feedback focus group discussions in Peru, Ecuador, and Brazil in July 2022. Third, the surveys were conducted and recorded in Spanish and translation errors may have affected our analysis. Finally, the interpretation of these narratives was subjective and affected by the researcher’s inherent biases. Again, the community engagement sessions helped to mitigate this bias by ensuring that the results resonated with Venezuelan migrants/refugees themselves. Nevertheless, using a sensemaking approach, our team was able to reach a large number of traditionally hard-to-reach participants across varied geographic locations.

## Conclusions

Pregnant Venezuelan migrants/refugees experience a series of additional vulnerabilities and encounter complex gender-based and societal issues that are rarely sufficiently reported on. Our data revealed 4 key themes: xenophobia while accessing maternal healthcare, GBV during and after migration route, lack of adequate financial resources, food and shelter and women leaving to better their children’s lives. Our study results can be used by international organizations and non-governmental organizations to advocate for these women and develop sustainable strategies to improve the experiences and outcomes of migrating pregnant women.

### Future directions

GBV and other challenges Venezuelan women face while migrating are multifactorial. Our study illustrates that pregnancy is an additional important risk factor. Women who are pregnant are especially vulnerable both physically and emotionally and require particular attention as GBV and other challenges such as discrimination or lack of financial support is compounded by pregnancy. We recommend the following to governments, as well as to non-governmental, international and social support organizations:

1. Address xenophobia at health-care centres in host countries. This would include development of policies, training, and regulation, in collaboration with health-care workers, administrative staff and policy makers.
2. Create support spaces for pregnant women who have experienced xenophobia or GBV.
3. Find additional and alternative ways to address the lack of shelter, food and water for migrants/refugees, particularly pregnant women, when they arrive at their host country.
4. Include pregnant women –and their specific needs– in regularization and socioeconomic integration initiatives, prioritizing safe, appropriate and reliable livelihood strategies.
5. Provide access to contraception and sexual health resources, including prenatal and postpartum care.
6. Promote solidarity and empathy within the healthcare system, as well as other public and private spaces. Public policies should be more inclusive and recognize the potential of refugees/migrants to contribute to their host community if provided with appropriate assistance and support.

Solutions implemented by organizations should be developed in partnership with and tailored specifically for pregnant women.

### Supplementary Information

**Supplementary material 1.**

## Data Availability

We are in the process of preparing the data for deposit into Queen’s open access repository, Dataverse, which is part of a larger open access repository, Borealis. Currently the data is being anonymized for deposit into an open access repository. Once the data is deposited anyone who wishes to can download and disaggregate the data for their retrospective analysis. If anyone would like an update on the availability of our dataset, please contact the corresponding author at: mzaman@qmed.ca.

## Abbreviations

IOM: International Organization of Migration
R4V Platform: The R4V Coordination Platform for Refugees and Migrants from Venezuela
GDP: Gross Domestic Product
OHCHR: The Office of the United Nations High Commissioner for Human Rights
GBV: Gender-based violence
