# Supplementary material for: Experiences of pregnant Venezuelan migrants/refugees in Brazil, Ecuador and Peru: a qualitative analysis
Source: BMC Pregnancy Childbirth. 2024 Feb 23;24:157. doi: 10.1186/s12884-024-06334-0 (PMC10885441; doi:10.1186/s12884-024-06334-0)
Supplement: Supplementary file 1 — Supplementary material 1. [file 12884_2024_6334_MOESM1_ESM.docx]

**Supplementary Materials I.** Survey questions with possible responses

| Question | Possible Responses |
| --- | --- |
| Micro-narrative prompts | |
| Share a story that exemplifies what is the biggest *advantage* or *disadvantage* to being a woman/girl when it comes to leaving Venezuela to come to a place like this. | Micro-narrative recorded by participant |
| Provide a story that illustrates the biggest *fear* or *dream*of women/girls who have left Venezuela. | Micro-narrative recorded by participant |
| Provide a story that illustrates how being a woman/girl most *increases* or *decreases* the risks faced by women/girls who have left Venezuela. | Micro-narrative recorded by participant |
| Triads | |
| The shared story was mostly about… | 1) Insecurity/violence;  2) Financial needs;  3) Lack of access to rights, goods, or services  or some combination thereof |
| What caused the events in the shared story? | 1) Being a woman or girl;  2) Lack of documentation;  3) Lack of assistance/services  or some combination thereof |
| What type of support or services were most needed by the woman/girl in the shared story? | 1) Improved security/protection;  2) Medical care/psychosocial support;  3) Basic necessities like food, water, shelter  or some combination thereof |
| What were the main barriers or facilitators to accessing care and services in the shared experience? | 1) Information or knowing where to go;  2) Financial resources;  3) Availability of assistance/services  or some combination thereof |
| If relevant, what was the primary impact of the experiences shared? | 1) Poor mental health;  2) Injury/illness;  3) Discrimination/isolation  or some combination thereof |
| Dyads | |
| In the story you shared, who had power and control? | 1) The woman or girl;  2) Others around her  or some combination thereof |
| The events in the story… | 1) Occurred because of leaving Venezuela;  2) Would have occurred anyway in Venezuela  or some combination thereof |
| The woman/girl in the shared story was… | 1) Provided with too many supports/services;  2) Provided with absolutely no supports/services  or some combination thereof |
| As a result of experiences in the shared story, is the woman/girl’s future… | 1) Extremely compromised;  2) Secured more than necessary  or some combination thereof |
| Star Question | |
| Think about the story you shared and select the choices that relate to the story. Drag the corresponding stars into the square placing them where they best represent your perspective. Leave any choice in the list that doesn’t apply to your experience.    Axes:  Y-axis: What was the impact on the girl’s/women’s physical and emotional well-being (nothing – a lot)  X-axis: What was the impact of this on the women’s/girl’s financial security (nothing – a lot) | 1) Informal work  2) Exchange of sex for goods, services, and protection  3) Taking care of family members  4) Accessing services  5) Transportation  6) Discrimination for being Venezuelan  7) Access to contraceptives, gynecological services, and family planning |
| Multiple Choice Questions About the Shared Experience | |
| Who is the story about (choose only 1)? | 1) About me  2) About someone in my family  3) Someone else I know  4) Something I heard or read about  5) Prefer not to say/not sure |
| How often does the situation in your story occur (choose only 1)? | 1) It is very rare  2) It happens from time to time  3) It is somewhat typical  4) It happens all the time  5) Prefer not to say/not sure |
| What is the emotional tone of this story (choose only 1)? | 1) Strongly negative  2) Negative  3) Neutral  4) Positive  5) Strongly positive  6) Prefer not to say/not sure |
| How does this story make *you* feel (choose up to 2)? | 1) Relieved  2) Embarrassed  3) Afraid  4) Ashamed  5) Happy  6) Disappointed  7) Angry  8) Hopeful  9) Frustrated  10) Helpless  11) Worried  12) Lonely  13) Sad  14) Prefer not to say/not sure |
| What was the age of the woman/girl in the story (choose only 1)? | 1) Under age 18  2) 19 - 30 years  3) 31 - 45 years  4) >  45 years  5) Prefer not to say/not sure |
| Relative to the wealth level of others in the community, was the woman/girl in your story (choose only 1)... | 1) Very Poor  2) Poor  3) Average  4) Wealthy  5) Very Wealthy  6) Prefer not to say/not sure |
| Which of the following groups, if any, did the woman/girl in the story identify with and was most relevant to her experience shared in the story (choose only 1)? | 1) Having a disability  2) Experiencing mental health problems  3) Use of alcohol and drugs  4) Did not identify with any of these groups |
| Did the women or girl in the shared story identify as LGBTQ+ (choose only 1)? | 1) Yes  2) No  3) Prefer not to say/not sure |
| Which of the following groups, if any, did the woman/girl in the story identify with and was most relevant to her experience shared in the story (choose only 1)? | 1) Mestiza  2) Afro-descendant  3) Indigenous  4) Prefer not to say/not sure  5) Did not identify with any of these groups  6) Other: _______________ |
| How did COVID-19 impact the woman/girl’s experience in the shared story (choose only 1)? | 1) COVID-19 made the situation much worse  2) COVID-19 made the situation a little worse  3) COVID-19 had no impact on her situation  4) COVID-19 made the situation a little better  5) COVID-19 made the situation much better  6) Prefer not to say/not sure |
| Was contraception mentioned in this story? | 1) Yes  2) No  3) Not sure |
| Was there a pregnancy in this story? | 1) Yes  2) No  3) Not sure |
| Multiple Choice Questions About the Respondent | |
| What is your age? | Participant enters age in free text field |
| How do you identify (choose only 1)? | 1) Woman  2) Man  3) Non-binary  4) Not sure / prefer not to say |
| Do you identify with any of the following groups? | 1) LGBTQ+  2) Indigenous  3) Afro-descendant  4) Prefer not to say |
| How many children do you have (choose only 1)? | 1) 0  2) 1 – 2  3) 3 or more  4) Prefer not to say |
| What is your marital status (choose only 1)? | 1) Married  2) In a union  3) Divorced/Separated  4) Widowed  5) Single, never married  6) Prefer not to say |
| How long ago did you leave your home in Venezuela (choose only 1)? | 1) < 1 year  2) 1 – 3 years  3) 3 – 5 years  4) > 5 years  5) Prefer not to say/not sure |
| Where are you from (choose only 1)? | 1) Caracas D.F.  2) Amazonas  3) Anzoátegui  4) Apure  5) Aragua  6) Barinas  7) Bolívar  8) Carabobo  9) Cojedes  10) Delta Amacuro  11) Falcón  12) Guárico  13) La Guaira  14) Lara  15) Mérida  16) Miranda  17) Monagas  18) Nueva Esparta  19) Portuguesa  20) Sucre  21) Táchira  22) Trujillo  23) Yaracuy  24) Zulia  25) Prefer not to say |
| At this time, I am able to cope with the challenges I face: | 1) All the time  2) Most of the time  3) Some of the time  4) Never  5) Prefer not to say |
| I have access to things that make me happy: | 1) All the time  2) Most of the time  3) Some of the time  4) Never  5) Prefer not to say |
| Where was this micronarrative collected (choose only 1)? | 1) Tumbes  2) Lima  3) Tacna  4) Tulcán  5) Manta  6) Huaquillas  7) Boavista  8) Pacaraima  9) Manaus |
| What story number is this for the participant? | 1st  2nd  3rd  4th |
| Comments or anything else you would like to share | Free text field |
| *Response was optional for all questions. | |
